# Skin ultrasonography and magnetic resonance; new clinical applications and instrumentation

**DOI:** 10.1111/srt.13633

**Published:** 2024-02-23

**Authors:** Alexander Zemtsov

**Affiliations:** ^1^ University Dermatology Center Muncie Indiana USA; ^2^ Department of Dermatology Indiana University School of Medicine Indianapolis Indiana USA

**Keywords:** instrumentation and application, skin magnetic resonance, skin radiation, skin ultrasoundography

## Abstract

**Background:**

Technological advances in skin ultrasonography and magnetic resonance are discussed.

**Methods:**

Literature review.

**Results:**

40 publications cited.

**Conclusion:**

This article illustrates crucial contributions made by the Editors, the Editorial Board and this Journal to these fields.

## 1 INTRODUCTION

### 1.1 Skin ultrasonography historical note

High frequency skin ultrasonography (HFUS) was initially developed by Alexander and Miller to measure skin thickness^1^ (A scan). Professor Serup, a former Editor in Chief of this Journal, and his group were instrumental in development of HFUS B mode (two dimensional) scans for evaluation of variety of dermatological conditions.^2^ Soon thereafter in Europe skin ultrasound society was formed that was later renamed and became a skin imaging society and in 2005 it had merged with skin bioengineering society (its former principal officers Hoffman and el Gammal are on the Editorial Board of this Journal). Zemtsov, a present Editor in Chief of this Journal, was most likely the first US physician to gain competence in HFUS and in 1995 was asked to write and published an Invited Discussion/Editorial on this topic in Annals of Plastic and Reconstructive Surgery.^3^ Dr Wortsman, who is on the Editorial Board of this Journal, is at the present time considered a leading expert in the field of skin ultrasonography.^4^

### 1.2 Ultrasound instrumentation

For many decades two relatively small companies Cortex (DermaScan unit, Denmark) and Taberna Pro Medicum (DUB unit, Germany) had dominated the field of HFUS. DermaScan and DUB machines provide HFUS of 20 MHz and higher (up to 100 MHz). New machines in addition to HFUS also have Doppler mode for blood vessel visualization and mapping, advanced specialty software, iCloud storage, telemedicine and Ios & Android capability, and a high‐definition imaging at a fraction of the cost. Zemtsov, being a board‐certified Mohs surgeon with extensive expertise in Superficial Radiation Therapy (SRT) uses in his practice Clarius L20 HD_3_ Ultra‐High Frequency Linear Scanner. This machine has automatic depth adjustable frequency between 8 and 20 MHz and a maximum penetration/visualization depth of 4 cm.

### 1.3 New diagnostic ultrasound application

Yu and coworkers reported that HFUS image guided SRT (IG‐SRT) is superior to non‐image guided SRT.^5^, ^6^ Yu et al. reported 99.3% cure of cutaneous squamous cell carcinoma (SCC) and basal cell carcinoma (BCC) utilizing IG‐SRT in a study of 2917 patients.^5^, ^6^ Furthermore, Zemtsov while proposing Appropriate Use Criteria (AUC) for SRT emphasized in his recommendations the importance of utilization of HFUS while performing SRT.^7^ 99.3% IG‐SRT cure rate for BCC and SCC is at least as high as can be achieved with the treatment of these cancers with Mohs surgery.^5^, ^6^ Zemtsov has been treating with IG‐SRT and SRT at least a thousand patients at University Dermatology Center (UDC) offices. IG‐SRT allows at initial consultation correctly to determine tumor depth and thus to determine an appropriate D_50_ for a correct level of treatment energy and for a correct placement of radiation fields. During IG‐SRT therapy again HFUS allows for SRT tumor response monitoring and to maintain an appropriate placement of radiation fields. Finally, after proposed SRT AUC were published^7^ Zemtsov and the coauthor Cognetta, who is also an Associate Editor of this Journal and the SRT expert, were contacted by individuals who had a concern with our recommendation that SRT should be only administered by board certified Mohs surgeons and radiation oncologists. In all fairness Zemtsov was taught SRT by late professor Neldner who was a medical dermatologist (before Zemtsov started teaching SRT to dermatology residents). Therefore, the time will determine appropriate qualifications for SRT and IG‐SRT administration; at UDC Mohs surgeons, radiation oncologists and radiation therapists work as a collaborative team in administering IG‐SRT.

The Doppler feature in HFUS to visualize blood vessels opened numerous new possibilities and clinical applications for dermatologists, plastic, cosmetic, and ENT surgeons. HFUS is now used as an imaging guide to visualize supratrochlear and supraorbital arteries during filler injections in a glabellar area and thus prevent inadvertent administration of the filler material into these vessels that can lead to potentially catastrophic results such as a blindness.^8^ HFUS is now used both to make sure that paramedian forehead interpolation flap contains a supratrochlear artery and there is an adequate blood flow in this flap prior to the pedicle division.^9^ Skin ultrasonography is also utilized for an exact administration of hyaluronidase to remove an excessive and/or inappropriately placed hyaluronic acid cosmetic filler; HFUS also prevents inadvertent arterial hyaluronidase injection during this procedure.^10^ Furthermore, HFUS is used to detect hyaluronic acid granulomas.^11^ Finally, HFUS is useful to guide to prevent parotid gland damage during cosmetic thread facial lifting and to diagnose a damage to either a Stensen's duct or a parotid gland that occurs during these procedures.^12^

### 1.4 Therapeutic ultrasonography

Newly developed high frequency and high‐intensity focused ultrasound (HIFU) showed efficacy in tattoo removal; both as an adjuvant therapy to laser surgery and as a primary treatment modality for this indication.^13^ HIFU also appears to have potential as a therapeutic modality in removal of warts^14^ and as a skin rejuvenation treatment.^15^

### 1.5 Cutaneous magnetic resonance historical note

Zemtsov, who has a graduate degree in Magnetic Resonance is credited in obtaining first skin MRI images of skin lesions; both melanocytic^16^ and non‐melanocytic ones.^17^ The MRI images showed excellent correlation with histological findings, such as lesions depth.^17^ Furthermore, Zemtsov obtained first in vivo Magnetic Resonance spectra (MRS).^18^, ^19^ Based on these findings he was asked and published on this topic an Editorial in Archives of Dermatology^20^ {now JAMA Dermatology). These results and subsequent publications by other physicians resulted in all medical insurances in the US to cover MRI scans of skin malignant tumors. Finally, while performing MRS studies Zemtsov discovered phosphocreatine in a cutaneous tissue; a molecule that plays a crucial role in skin bioenergetic pathways.^18^, ^19^ Bernard Querleux, PhD, who is an Associate Editor of this Journal, was also a pioneer in skin MRI, MRS, and ultrasonography fields.^21^, ^22^

### 1.6 MRI and MRS instrumentation

To obtain both human in vivo MR images or MR spectra the same scanners containing superconducting magnets are used (the MRS requires a purchase of a separate software and it's primarily available only at academic institutions}. Initial MR scanners had magnetic field strength of 1.5 T (tesla)‐ newer machines have a strength of 3T.

In 1997 Zemtsov at a joint ISSI/ISDIS Congress in Vienna Austria suggested a development of a portable MRI machine utilizing permanent (instead of superconducting} magnets. 25 years later portable MRI is both a reality and is approved for clinical use‐ Swoop Portable MRI (produced by Hyperfine, Inc).

### 1.7 New MRI/MRS applications

The purpose of skin MRI (like general radiology applications) is to measure preoperatively tumor depth, to assess an extent of underlying tissue involvement (such as orbit involvement etc.), and to evaluate tumor recurrence under skin flaps or grafts.^16^, ^17^, ^20^, ^23^ MRI can be also used to monitor tumor response to therapy and to determine the type of malignancy present.^20^ Nowadays it became apparent that skin MRI is a technique of choice to discover the presence of a perineural involvement in high‐risk cutaneous squamous cell carcinomas^24^ (especially when a patient exhibit symptoms suggestive of cranial nerve involvement).^24^

Kawaguchi and coworkers recently confirmed Zemtsov findings^16^ that skin melanoma produce hyperintensive T1 images^25^ (due to paramagnetic properties of melanin that acts as MRI contrast material). The same research group confirmed another Zemtsov suggestion^17^, ^20^ that certain radiological criteria are more characteristic in MRI images of basal cell versus squamous cell carcinomas^26^ (ulceration and protrusion into the fat in SCC and hyperintense T2 images in BCC). Furthermore, recent reports demonstrated the utility of MRI in evaluating dermatofibrosarcoma tumors^27^ as with tumors mentioned above in this paragraph MRI was useful in designing a surgical excision and in differentiating between this tumor and other lesions.^27^

New skin/skin related MRI diagnostic applications include MRI evaluation of scalp alopecia^28^, ^29^ (primary use is to evaluate effectiveness of new therapies‐ see MRS discussion below), abnormal brain MRI findings in patients with atopic deramtitis^30^ and use of brain functional MRI to evaluate changes induced by skin pricking/itch/pain sensation.^31^, ^32^

In the field of skin MRS no new studies appeared recently. Zemtsov demonstrated a potential utility of this technique to evaluate efficacy of new psoriasis therapies^33^; Zemtsov also suggested MRS can be used in development and evaluation of sunscreens^34^ ‐see MRI of alopecia discussed in the previous paragraph. Finally, Zemtsov revealed that MRS can be used to monitor progress in wound healing of leg ulcers^35^ (MRS requires specialized training in medical physics beyond what is normally available during radiology or radiation oncology residency training). Nevertheless, subsequent investigations confirmed Zemtsov discovery of phosphocreatine in a cutaneous tissue explaining a unique ability of a human skin to withstand ischemia associated with skin flaps, skin grafts, and hair transplantation procedures.^36^, ^37^

### 1.8 Therapeutic magnetic fields and thermal MRI

Spinning oscillating magnetic fields induce within malignant cells oxygen radical molecules causing cellular apoptosis.^38^ This is a mechanism of a new investigational device called “oncomagnetic helmet” for treatment glioblastoma brain tumors^39^ (technically this clearly is not a magnetic resonance phenomenon since no Larmour frequency is applied to generate quantum states). Both in the US and Europe there are efforts to develop a Thermal MRI machine that in addition to diagnostic information will also generate radiofrequency induced tumor ablating heat in MRI guided area^40^ (for example it may allow neuroradiologists, utilizing Thermal MRI, instead of neurosurgeons to treat glioblastomas).

## 2 CONCLUSION

This review summarizes the advances in Skin Ultrasonography and Magnetic Resonance. Furthermore, this article illustrates crucial contributions made by the Editors, the Editorial Board and this Journal to these fields.

## Data Availability

The data that support the findings of this study are available in PubMed at https://pubmed.ncbi.nlm.nih.gov/. These data were derived from the following resources available in the public domain:—https://pubmed.ncbi.nlm.nih.gov/, https://pubmed.ncbi.nlm.nih.gov/
